# Predictive value of electrocardiographic markers in children with dilated cardiomyopathy

**DOI:** 10.3389/fped.2022.917730

**Published:** 2022-08-23

**Authors:** Miao Wang, Yi Xu, Shuo Wang, Ting Zhao, Hong Cai, Yuwen Wang, Runmei Zou, Cheng Wang

**Affiliations:** ^1^Department of Pediatric Cardiovasology, Children’s Medical Center, The Second Xiangya Hospital, Central South University, Changsha, China; ^2^Department of Neonatology, Xiangya Hospital, Central South University, Changsha, China

**Keywords:** dilated cardiomyopathy, prognosis, children, electrocardiography, marker

## Abstract

Dilated cardiomyopathy (DCM) refers to a heterogeneous group of cardiomyopathies characterized by ventricular dilatation and myocardial systolic dysfunction, which can lead to serious consequences such as malign arrhythmia, sudden death, heart failure, and thromboembolism. With its economical, non-invasive, simple and reproducible advantages, electrocardiogram (ECG) has become an important indicator for assessing the prognosis of cardiovascular diseases. In recent years, more and more studies of electrocardiography on DCM have been carried out, but there is still a lack of a comprehensive summary of its prognostic value. This article reviews the prognostic value of electrocardiographic markers in children with DCM.

## Introduction

Dilated cardiomyopathy (DCM) is a type of cardiomyopathy with left or biventricular dilatation and systolic dysfunction (left ventricular ejection fraction (LVEF) ≤35%) in the absence of coronary artery disease, hypertension, valvular disease, or congenital heart disease. Various etiologies lead to DCM, including infections, genetic mutations, autoimmune diseases, inflammation, exposure to toxins, and endocrine or neuromuscular causes. DCM is the most common form of cardiomyopathy in children with an annual incidence of 0.18∼0.73 per 100,000 person-years and usually leads to adverse end-point events involving malign arrhythmia (MA), sudden death (SD), heart failure (HF) and thromboembolism (1, 2). In the last 30 years, the improvement of early diagnosis and treatment levels has significantly contributed to improvement of survival, but risk stratification and prognosis prediction remain a challenge (3). The electrocardiogram (ECG) of DCM may be normal, but many patients also undergo abnormalities including isolated T-wave changes, left bundle branch block, prolonged atrioventricular conduction, and supraventricular arrhythmia (1). Disbalance of the autonomic nervous system and impaired uniformity of myocardial repolarization are two major underlying mechanisms for developing ventricular arrhythmias in patients with DCM (4, 5). With the increasing research on ECG in patients with DCM, ECG abnormalities may reflect the severity of cardiovascular disease and provide a simple and useful tool for risk stratification and prognosis prediction; this article reviews the prognostic value of ECG markers in pediatric DCM.

## P wave

The front one-third of the P wave is the right atrial depolarization vector, the middle one-third is the comprehensive vector of right and left atrial depolarization, and the last one-third stands for the left atrial depolarization vector. The addition of the algebraic sum of positive and negative P wave amplitudes (ΣP amplitude) in 12-lead ECG is strongly associated with atrial enlargement caused by DCM (6).

Prolonged P wave duration (P > 120 ms) is an index of left atrial abnormality, reflecting electromechanical dysfunction, and structural remodeling of the atrium, and is associated with myocardial fibrosis, atrial fibrillation, and sudden cardiac death (SCD). Studies have demonstrated that P-wave duration is significantly correlated with the atrial size (7). P-wave dispersion (Pd) is the difference between the maximum and minimum P-wave duration on surface ECG. Pd is a new maker of abnormal atrial conduction in ECG leads in different directions, reflecting inhomogeneous and slow atrial conduction. Increased Pd is a reliable predictor of atrial fibrillation and is associated with SCD and severe ventricular arrhythmia (4, 8, 9). Türe et al. (4) divided 85 children with DCM into two groups, surviving patients (73 cases with mean diagnosis age of 32 ± 45.6 months) and exitus patients (12 cases with mean diagnosis age of 58.88 ± 72.3 months); a significant difference in Pd was found between the two groups through ECG data (40 ± 15 ms vs. 56 ± 23 ms, *P* < 0.05); Pd was significantly increased in the death group. These results suggested that abnormal atrial conduction may lead to SD in patients with DCM.

P-wave terminal force in lead V1 (PTFV1) is the product of the negative terminal P-wave amplitude and the negative P-wave time in lead V1. Increase of cardiac load, myocardial hypertrophy, myocardial fibrosis, and conduction delay can lead to abnormal PTFV1 (10–13). The negative increase of PTFV1 in patients with DCM is related to LVEF. The proportion of PTFV1 ≤ 0.03 mm⋅s has significant differences in patients with LVEF <40%, LVEF 40-45%, and LVEF >45% (78.8% vs. 60.1% vs. 9%, *P* < 0.01), suggesting that PTFV1 is helpful in judging the functional status of the left heart in DCM (11). Deep terminal negativity of P wave in V1 (DTNPV1) was defined from the resting 12-lead ECG as the presence of biphasic P wave (positive/negative) in V1 with the amplitude of the terminal negative phase > 100 μV, or one small box on ECG scale (14). Larisa et al. (14) adjusted risk stratification for 15,375 participants (mean age 54.1 ± 5.8 years) and found that the risk of SCD associated with DTNPV1 was still significant (*P* < 0.01), indicating that DTNPV1 has potential application value in the SCD risk stratification of the general population. Although there are few studies about PTFV1 and DTNPV1 in children, the previous large sample size study in adults has a reference value and provides new ideas for related research in children.

## QRS wave

### QRS amplitude

The R+S amplitude on ECG is the sum of the projected amplitudes of the spatial QRS vector loop on the positive and negative sides of the corresponding lead axis. The amplitude of R_*v1*_+S_*v5*_, reflecting the right ventricular depolarization potential, is a common indicator for the diagnosis of right ventricular hypertrophy with high sensitivity but low specificity. The amplitude of R_*I*_+S_*III*_, reflecting the comprehensive vector of leftward and upward depolarization of the ventricle, is a common maker for the diagnosis of left ventricular hypertrophy (6).

Previous studies have shown that increased ΣQRS (measured by the Siegel method) has high sensitivity and specificity for the diagnosis of left ventricular hypertrophy (15). Studies have shown that high QRS voltage is usually observed in patients with LVH and generally can increase mortality and predict the risk of progression of HF (16, 17). Increased QRS amplitude in lead aVR is associated with inverse left ventricular remodeling, and QRS amplitude in the precordial and right-sided chest leads may reflect both myocardial viability and cardiac size in patients with non-ischemic cardiomyopathy (18).

Reduced QRS amplitude is also associated with severe outcomes in patients with DCM. Merlo et al. (19) found that the decreased S wave amplitude in lead V2 (*P* < 0.01) and the decreased R wave amplitude in lead III (*P* < 0.01) were associated with myocardial fibrosis, and could predict the risk of SCD and malignant arrhythmia in patients with DCM. Although this study is not specific for children, the conclusions obtained after long-term follow-up (average 125 months) provide valuable information for related research in children. Low QRS voltage may reflect a decrease in electrical activity throughout the heart and is associated with clinical deterioration and poor prognosis in left ventricular systolic dysfunction. The QRS amplitudes were decreased with the deterioration of HF and increased with the recovery of the HF. The main mechanism of the change in the QRS amplitudes may be related to the increase or decrease of the interstitial fluid in patients with decompensated HF (DHF) (20, 21). Das et al. (22) found that each abnormally low-amplitude intra-QRS peak was associated with a 2.1-fold increased arrhythmic risk (*P* < 0.01). The QRS peak is a novel indicator that involves automatic quantification of abnormal, low-amplitude intra-QRS peaks from high-resolution ECGs, which has a research value in children with DCM. The mechanism may be due to inhomogeneous scarring in the two ventricles, producing variable transient electrical vectors, resulting in the occurrence of potentials within the QRS that promote ventricular tachycardia (VT) through slow or tortuous conduction, or simple time-varying conduction, finally inducing ventricular tachyarrhythmias.

### QRS duration

QRSd stands for ventricular depolarization time. A prolonged QRSd indicates an increase in the heterogeneity of ventricular depolarization, suggesting that patients may have left ventricular dysfunction, myocardial hypertrophy, structural changes in the cardiac conduction system, or cardiac asynchrony, which can predict adverse clinical conditions such as HF and SCD (18, 23, 24). QRSd prolongation occurs in 14%–47% of HF patients, and QRSd prolongation is an independent risk factor for the induction of VT (25, 26).

Progressive myocardial fibrosis can lead to asynchronous activation of left and right ventricular and QRSd prolongation (27). Dao et al. (28) studied the ECG of 183 children with DCM (age 7.9 ± 6.6 years) and found that DCM children developing life-threatening arrhythmias had a longer QRSd Z score (4.9 vs. 2.2, *P* < 0.05). In the ROC curve with the true-positive rate (sensitivity) and false-positive rate (1-specificity) of the QRS duration Z-score value as the axes, the area under the curve (AUC) was 0.78, suggesting that prolongation of QRSd is a significant risk factor of life-threatening arrhythmia. Türe et al. (4) divided 85 children with DCM into two groups, the surviving patients (73 cases with the mean diagnosis age of 32 ± 45.6 months) and the exitus patients (12 cases with the mean diagnosis age of 58.88 ± 72.3 months), the mean QRSd was significantly longer in the exitus children than in the surviving children with DCM (66 ± 13 ms vs. 82 ± 34 ms, *P* < 0.05). Choi et al. (29) reported that 97 (38%) of 253 patients with non-ischemic DCM (NIDCM) with left ventricular reverse remodeling (LVRR), among which patients with QRSd > 120 ms, the ratio of LVRR to No LVRR was 13.4% vs. 35.9% (*P* < 0.01), suggesting that QRSd may be related to the severity of myocardial structural changes. LVRR in NIDCM is more common in patients with normal QRSd besides associated with a favorable prognosis in these patients. However, other studies have demonstrated that narrow QRSd (QRSd < 120 ms) is also associated with intra-ventricular dyssynchrony, which may be due to fibrotic myocardial tissue triggering the dyssynchrony of myocardium in patients with DCM. Approximately 27–56% of patients with narrow QRSd exist intra-ventricular dyssynchrony and are probably suggestive of a poor prognosis (30, 31). Andrea et al. (31) reported 180 patients with idiopathic DCM (LVEF ≤ 35%, QRSd <120 ms); all the patients underwent standard Doppler ultrasound, color tissue velocity imaging (DTI), and supine bicycle exercise stress echocardiography. The increase of intra-ventricular delay ≥30 ms was found to have a sensitivity and specificity of 86.7% and 78.8% for predicting adverse cardiac events (AUC: 0.88, *P* < 0.01), indicating that intra-ventricular dyssynchrony in patients with QRSd <120 ms is associated with poor prognosis. It is well known that the QRSd in children is slightly shorter than that in adults, usually between 40 ms and 80 ms. Previous studies have shown that both the prolongation and shortening of QRSd are related to intra-ventricular dyssynchrony. The specific reference value in children still needs further research. As mentioned above, QRSd has an important value in the prognosis prediction in children with DCM and is mainly related to fatal arrhythmias caused by asynchrony of cardiac electrical activity.

### Fragmented QRS

Fragmented QRS is a simple indicator of myocardial fibrosis on the surface ECG, which included various morphologies of the QRS. Narrow fQRS (QRS < 120 ms) refers to an additional R’ wave in two contiguous leads or notching in the nadir of the S’ wave; wide fQRS (QRS ≥ 120 ms) is > 2 R’ or 2 S’ waves in two contiguous leads (32, 33). The presence of fQRS is associated with altered myocardial activation due to myocardial scarring and fibrosis. When the myocardium undergoes fibrosis and scar repair, the activation of ventricular myocardium is delayed, and the activation heterogeneity of various parts increases significantly, which is manifested as the formation of QRS notch on the surface ECG (32).

Fragmented QRS may predict adverse outcomes such as severe arrhythmia, SCD, and HF, and associated with LV dyssynchrony in idiopathic DCM patients and can be used as a reliable ECG marker for evaluating left ventricular dyssynchrony and predicting prognosis in patients with idiopathic DCM (34). Kong et al. (35) divided 63 children (31.2 ± 74.2 months) with IDCM into the non-fQRS group (*n* = 38) and fQRS group (*n* = 25); the study found that the LVEF of the fQRS group was lower than that of the non-fQRS group (23.9% ± 8.3% vs. 29.9% ± 10.5%, *P* < 0.05), the QRSd was also prolonged (98.8 ± 27.0 ms vs. 80.6 ± 20.2 ms, *P* < 0.01), and the incidence of arrhythmias was higher (36.0% vs. 7.9%, *P* < 0.01), suggesting that the positive fQRS complex in children with DCM was identified as risk factors for adverse outcomes. Besides, the age of fQRS group was significantly older at diagnosis (86.6 months vs. 44.1 months, *P* < 0.05). These results demonstrated that fQRS is a strong predictor of adverse cardiovascular events. However, Cheema et al. (36) reported that the presence of fQRS on the ECG was not associated with a higher risk of all-cause or arrhythmic death (*P* = 0.38) after following up 842 patients with left ventricular dysfunction. Although the age group included in this study is over 20 years old, it also provides a new idea for fQRS predicting the prognosis of DCM in children. Konishi et al. (18) found that in patients with non-ischemic cardiomyopathy; fQRS was associated with a reduced incidence of LVRR (*P* < 0.01), but not with LGE (*P* = 0.49). Multiple studies have shown that fQRS is associated with myocardial fibrosis, but there is no consensus on whether fQRS is associated with worse prognosis in patients with DCM.

### QRS-T angle

The frontal QRS-T angle is defined as the angle between the depolarization and repolarization directions of the ventricle and can be easily obtained from the frontal QRS and T axes. The increased QRS-T angle reflects both structural abnormalities affecting depolarization and pathophysiological changes in ion channel regions that alter the repolarization order (37–39).

Abnormal QRS-T angle (>67°) can predict the risk of death (*P* < 0.01) (40). Li et al. (41) studied 509 patients with idiopathic DCM and showed that the QRS-T angle was independently associated with all-cause mortality (*P* < 0.05), cardiac mortality (*P* < 0.05), and HF rehospitalization (*P* < 0.01). The optimized treatment significantly reduced the frontal QRS-T angle (100.9 ± 53.4° vs. 107.2 ± 54.4°, *P* < 0.01). Aro et al. (42) reported 10,957 middle-aged subjects and found that the frontal QRS-T angle ≥100° accounted for 2.0%, which was associated with an increased risk of sudden arrhythmic death (*P* < 0.01) and all-cause mortality (*P* < 0.01), but not non-arrhythmic cardiac death (*P* = 0.13). The spatial QRS-T angle is the angle between the spatial QRS maximal vector and the spatial T maximal vector, as well as the angle between the directions of ventricular depolarization and repolarization, usually used to predict mortality from ischemic cardiomyopathy in the general population (43). The above studies included a large number of samples, but unfortunately, there is a lack of specific studies for children. The spatial vector can be measured by new electrocardiographic techniques, which provides a new tool for prognosis prediction in children with DCM.

### QT interval and QTd

QT interval represents the period from the beginning of QRS wave to the end of the T wave, which reflects the length of the ventricle from depolarization to the end of repolarization, shortened or prolonged QT interval on the ECG indicates changes in repolarization time and is the electrophysiological basis for fatal ventricular arrhythmias (44). The corrected QT interval (QTc) is based on the Bazett formula (45): QTc = QT/$\sqrt{R\,R}$, which is corrected for heart rate and often used in studies to predict mortality.

QTc prolongation is often associated with severe arrhythmia evens in children with DCM. Chen et al. (46) reported 137 children (mean age 7.8 ± 6.7 years) with DCM (overall mean QTc was 456 ± 52 ms) and found that children with life-threatening ventricular arrhythmia evens had a longer QTc (488 ± 96 ms vs. 453 ± 44 ms, *P* < 0.05), suggesting that QTc prolongation (≥510 ms), characteristics of ECG repolarization, is strongly associated with life-threatening arrhythmia evens. Sabaz et al. (47) reported 37 children with DCM, 21 boys (56%), and 16 girls (44%), with an average age of 27.50 ± 50 months and 67.6% of the children were diagnosed with DCM at the age <12 months. They divided the children into non-survival group and surviving group and after comparing the presentation of 12-lead surface ECG between the two groups, they found that the non-survival group had a longer QTc than the surviving group (390∼410 ms vs. 260∼410 ms, *P* < 0.01), which suggested QTc to be a predictor of mortality in children with DCM. Ryerson et al. (48) reported 38 children (4.5 ± 5.4 years) with DCM, after 50 months of follow-up, the survival probability of children with longer QTc was lower than that of children with normal QTc (50% vs. 72%, *P* < 0.01), indicating that the QTc of the deceased children was significantly prolonged (*P* < 0.01). However, Pahl et al. (49) reported a study of 566 children with DCM although only 5 of the 35 children with SCD had quantitative ECG data, the mean QTc in these five children was very similar to that in the 531 children without cardiac death (449 ± 72 ms vs. 428 ± 50 ms), but data were not used in multivariate analysis. Therefore, most studies have shown that QTc prolongation has a great predictive value for worse prognosis in children with DCM and is mostly associated with ventricular arrhythmia evens.

QTd is the difference between the longest QT interval and the shortest QT interval on surface 12-lead ECG, representing the repolarization asynchrony and electrical instability of the ventricular myocardium in different parts. The increase of QTd represents that the difference of repolarization of ventricular myocytes is increased, as a result, and is easy to cause abnormal conduction to form reentry, which can induce malignant arrhythmia or SCD (50, 51). Türe et al. (4) studied the ECG results of 85 children with DCM (12 of them died) and found that the survival group was significantly better than the death group in terms of QTd (42 ± 20 ms vs. 70 ± 45 ms, *P* < 0.01) and corrected QT interval dispersion (QTcd) shortening (38 ± 27 ms vs. 74 ± 77 ms, *P* < 0.05), suggesting that prolonged QTd and QTcd are associated with mortality in children with DCM. However, Fauchier et al. (52) reported 162 adult patients with DCM (age 52 ± 12 years) and found no difference in QTd between the event-free group (*n* = 113) and the cardiac death group (*n* = 26) (435 ± 42 ms vs. 442 ± 39 ms, *P* = 0.71), as well as the event-free group (*n* = 113) and the arrhythmia group (*n* = 23) (435 ± 42 ms vs. 440 ± 41 ms, *P* = 0.60), which suggested that QTd has no value for cardiac risk stratification. Therefore, the relationship between the estimated value of QTd for poor prognosis and the age of patients with DCM deserves further study.

## ST segment

ST segment is the segment from the end of the QRS to the beginning of the T wave, which is equivalent to the two-phase plateau in the action potential. In general population, ST segment depression in limb leads is rare, except for aVR lead, which is 12%, all other leads are <5% from neonate to 84 years old. ST-segment elevation in precordial leads is more common than in limb leads. ST-segment elevation in limb leads is often <0.1 mV except in lead aVL. Not only the occurrence rate of ST-segment elevation is high but also the amplitude of the elevation is large in the precordial leads (53).

In children with DCM, the ST segment in the limb leads moved down, the ST segment in the right chest leads was elevated, and the ST segment in the left chest leads was significantly decreased. The incidences of ST segment depression in leads I, II, III, aVF, V4, V5, and V6 were, respectively, 32.69, 42.31, 61.54, 50.00, 32.69, 50.00, and 65.39% (*P* < 0.01), illustrating myocardial ischemia (6). Sabaz et al. (47) studied the ECG data of 37 patients (27.50 ± 50 months) with DCM (six of them died) and found that the ST-T wave change rate in the death group was higher than that in the survival group (80 vs. 17.8%, *P* < 0.05). Chen et al. (46) conducted a survival analysis of 137 children with DCM (aged 7.8 ± 6.7 years) and showed that 2 of the 15 children with fatal arrhythmia evens had ST-segment depression (depression >1 mV in two consecutive leads), 6 of 122 children without fatal arrhythmia had ST segment depression, demonstrating that ST segment depression is a risk factor for fatal arrhythmia evens (*P* < 0.05). Takahama et al. (54) analyzed 12-lead ECG in cardiopulmonary exercise testing in 360 patients with non-ischemic DCM and discovered that the event-free survival rates for major cardiac events in patients with or without exercise-induced ST-segment elevation (ESTE) were 61 and 88%, respectively, after 48 months follow-up, indicating that ESTE was the strongest independent prognostic indicator among exercise parameters (*P* < 0.05), possibly reflecting the pathophysiological process of worsening HF. ESTE is an emerging parameter that has a good predictive value for exercise tolerance in patients with DCM, but the current study is not specific to pediatrics, and further large-sample studies are needed to confirm its predictive effect.

## T wave

T wave represents the process of ventricular repolarization, which is equivalent to the three-phase action potential. The ischemic T wave is inverted in the ECG; the changes in T wave amplitude and Tp-Te interval are of great significance for predicting SCD, MA, and other adverse prognoses.

### T wave inversion

When the subepicardial ischemia occurs, the duration of the epicardial action potential is significantly prolonged, and the repolarization sequence is reversed. The T wave representing the repolarization vector is opposite to the repolarization direction, so that the lead facing the ischemic area records an inverted T wave (55).

T-wave inversion may signal severe arrhythmia. Chen et al. (46) reported the ECG of 137 children (7.8 ± 6.7 years) with DCM. They found that the proportion of T-wave inversion was higher in children with life-threatening arrhythmia events than children without life-threatening arrhythmia events (27% vs. 8%, *P* < 0.05), indicating that T-wave inversion is closely related to life-threatening arrhythmia events. Survival analysis of all children with DCM with T wave inversion in ECG showed that the risk of death was increased by 10.6 times (HR = 11.6, *P* < 0.01). Merlo et al. (19) improved the ability to predict SD or malignant ventricular arrhythmia (SD/MVA) in patients with DCM when the ECG model was added to the clinical-echocardiographic model, the AUC was increased from 0.68 to 0.74 by ROC analysis (*P* < 0.05), further research found that the presence of T wave inversion in the anterolateral leads of the surface ECG in patients with DCM can be used as an independent predictor of SD/MVA (*P* < 0.05), which may be a sign of myocardial disorder and related to specific genotypes (i.e., intercellular connexin mutations) with potential overlapping phenotypes between DCM and arrhythmogenic right ventricular cardiomyopathy (ARVC). This study is significant and involves data related to gene mutation of cardiomyopathy. It is worth further predicting the prognosis of children with DCM through age stratification and etiological classification, and has a good research prospect.

### T-wave amplitude

A low level of T-wave amplitude is mostly indicative of myocardial ischemia. Tan et al. (55) reported the ECG results of 44 children with DCM. The results demonstrated that the T-wave amplitude in leads II, V4, V5, and V6 in the LVEF < 50% (8.4 ± 3.4 years) group was significantly lower than that in the LVEF ≥50% (7.5 ± 2.9 years) group, revealing that significant left ventricular myocardial ischemia may be related to the significant decrease of Ito, Ikr, and Ik1, the decreased potassium efflux, prolonged repolarization time, and increased action potential duration in HF. When T-wave amplitude in lead II ≤0.20 mV, T-wave amplitude in lead V4 ≤0.40 mV, T-wave amplitude in lead V5 ≤0.3 mV, and T-wave amplitude in lead V6 ≤0.30 mV, the sensitivity of predicting LVEF <50% in children with DCM was 88.2%, and the specificity was 76.0%.

### T-wave alternans

T-wave alternans is the alternating changes in the shape and amplitude of the ST segment or T wave between adjacent heartbeats on the ECG during sinus rhythm (the amplitude difference in any lead is ≥ 0.1 mV). Most of them are microvolt TWA (MTWA), reflecting the spatiotemporal heterogeneity of repolarization. TWA refers to the alternation of action potential time course between each beat at the level of cardiomyocytes. This repolarization heterogeneity can be either coordinated alternation or uncoordinated alternation. When uncoordinated electrical alternation occurs between adjacent cardiomyocytes, the discreteness of myocardial repolarization increases, then conduction block and reentry are formed between adjacent cardiomyocytes, resulting in malignant arrhythmia (56, 57).

In recent years, multiple studies have shown that TWA can be used as an ECG marker for fatal arrhythmia evens. Makarov et al. (58) studied 68 healthy children and 85 children with cardiovascular disease and found that TWA values did not exceed 55 μV in 94% of healthy children, but 20–50% of children with cardiac pathology had TWA values above 55 μV. Nocturnal circadian rhythm patterns exceeding 55 μV in TWA-associated diseases had risks of life-threatening arrhythmias. Furthermore, TWA is significantly different between DCM and healthy children (68 ± 43 μV vs. 30 ± 11 μV, *P* < 0.01). Salerno et al. (59) reported the same results in a study of 446 patients with NYHA functional class II/III non-ischemic cardiomyopathy, with a 3-fold increased risk of cardiac death and life-threatening arrhythmia evens in TWA-positive patients (6.5% vs. 1.6%, *P* < 0.01). However, there are contradictory results. Grimm et al. (60) reported 343 patients with idiopathic DCM and found that the positive TWA was not associated with an increased risk of arrhythmia (*P* = 0.75), which may be due to differences in methods or study populations and deserves further investigation. In the current research on the prognosis of DCM, there are relatively few data on TWA, but related studies in adults have revealed the close relationship between TWA and poor prognosis such as arrhythmia, thus providing more directions for pediatric research.

### Tpeak-tend interval

Tp-Te refers to the time from the peak to the end of the T wave in the ECG, in other words, the entire T wave descending branch time, which represents the relative refractory period of the ventricular myocardium and reflects the ventricular transmural repolarization dispersion.

Several previous studies have shown that Tp-Te and Tp-Te/QT ratio are ECG indicators for predicting malignant ventricular arrhythmia events and are closely related to the occurrence of various cardiovascular diseases contributing to SCD. Türe et al. (4) reported 85 children with DCM (12 cases died) and demonstrated that Tp-Temax in the survival group was shorter than that of the death group (74 ± 14 ms vs. 87 ± 23 ms, *P* < 0.05), the Tp-Te/QT ratio became smaller (0.037 ± 0.014 vs. 0.050 ± 0.018, *P* < 0.05), which indicated that prolonged Tp-Temax and increased Tp-Te/QT ratio were associated with enhanced mortality in children with DCM, but Tp-Temax/QTmax ratio (0.255 ± 0.073 vs. 0.272 ± 0.063, *P* = 0.53) and Tp-Temax/QTcmax ratio (0.174 ± 0.038 vs. 0.195 ± 0.055, *P* = 0.18) had no statistical difference. Shimizu et al. (61) found that Tp-Te (*P* < 0.01) was better than QTcd (*P* < 0.05) in predicting SCD / VT in patients with hypertrophic cardiomyopathy (HCM) associated with K183del mutation of cTnI gene. Whereas Porthan et al. (62) reported no association between Tp-Te prolongation and SCD (*P* = 0.231) in a prospective study of more than 5,600 cases that were followed up for nearly 8 years. This study included a large number of samples, but did not include DCM, so more data are needed to confirm the significance of Tp-Te in the prognosis prediction of DCM. Consequently, there is no consensus on the significance of Tp-Te in the prediction of malignant cardiac events.

## Conclusion

Dilated cardiomyopathy is a type of heterogeneous cardiomyopathy characterized by ventricular dilatation and decreased myocardial systolic function, commonly resulting in adverse outcomes involving SCD and HF often occurs in clinical practice. Pediatric DCM has a 5-year survival rate of 54% (63), accordingly, early evaluation of relevant indicators impacting its prognosis is of great significance. Numerous studies have shown that electrocardiographic indicators such as P wave, QRS wave, QT interval, and QT interval dispersion, ST segment, and T wave in the body surface ECG have a positive predictive value on the adverse prognosis of children with DCM including malignant arrhythmia, SCD and HF (Table 1), but the prognostic value of different ECG indicators is still somewhat controversial. Additionally, the study found that among Chinese children with DCM, when the combined index of T complex amplitude in lead aVR difference and QT interval difference in lead V6 were -0.05 mV and 5 ms, the sensitivity and specificity for estimating DCM prognosis were 44.4% and 83.3%, respectively. This may be related to various factors such as myocardial injury and autonomic dysfunction. Stimulation of the sympathetic nervous system induces a cardiac hypertrophic response (64). Although some parameters are less studied in DCM, and there is no age or etiology stratification for DCM, previous studies also provide ideas and directions for future research in DCM. Although the common 12-lead ECG has the advantages of convenience and speed, with the development of artificial intelligence, new ECG analysis techniques are playing an increasingly important role in the diagnosis, etiology analysis, and prognosis prediction of DCM. As prototype design, the wet Piotrode^®^ electrode can reduce electrode motion artifacts of electrostatic and mechanical origins and especially in QRS detection performance when the patient is exercising (65).

**TABLE 1:** Different electrocardiographic markers of adverse prognosis of children with DCM.

Markers	Classification	References	N	Cutoff values	*P*-value
P wave	Pd	Ture et al. (4)	85	56 ± 23 ms	< 0.05
	DTNPV1	Larisa et al. (14)	15375	> 100μV	< 0.01
QRS wave	QRSp	Das et al. (23)	99	≥ 2.25	< 0.05
	QRSd	Ture et al. (4)	85	66 ± 13 ms	< 0.05
	QRSd Z score	Dao et al. (31)	183	4.9	< 0.05
	fQRS	Kong et al. (38)	63	−	< 0.01
QRS-T	QRS-Tangle	Aro et al. (45)	10,957	≥ 100°	< 0.01
		Chaudhry et al. (47)	178	> 152°	< 0.01
QT	QTd	Fauchier et al. (57)	162	435 ± 42 ms	= 0.71
		Türe et al. (4)	85	42 ± 20 ms	< 0.01
	QTc	Chen et al. (50)	137	≥ 510 ms	< 0.05
		Karatolios et al. (53)	272	> 440 ms	< 0.01
	QTcd	Türe et al. (4)	85	38 ± 27 ms	< 0.05
		Shimizu et al. (67)	47	−	< 0.05
ST	ST-segment depression	Chen et al. (50)	137	> 1 mV in two consecutive leads	< 0.05
T	T wave inversion	Merlo et al. (20)	414	27%	< 0.05
	Tp-Te	Shimizu et al. (67)	47	−	< 0.01
	Tp-Temax	Türe et al. (4)	85	74 ± 14 ms	< 0.05
	Tp-Te/QT ratio	Türe et al. (4)	85	0.037 ± 0.014	< 0.05
	ESTE	Takahama et al. (59)	360	≥ 0.1 mV	< 0.05

Pd: P-wave dispersion; DTNPV1: Deep terminal negativity of P wave in lead V1; QRSp: intra-QRS peaks; QRSd: QRS duration; fQRS: fragmented QRS; QTc: the corrected QT interval; QTcd: corrected QT interval dispersion; Tp-Te: Tpeak-Tend Interval; ESTE: ST-segment elevation.

For example, combining primitive machine learning (ML) methods with protein–protein interaction networks to identify specific disease-associated molecular targets, i.e., preliminary specific genes, may lead to rapid selection of molecular targets associated with clinical parameters of DCM. Besides, ML technique could effectively predict adverse events in DCM patients with severely reduced LVEF and predicted risk in patients with severe DCM in 1-year follow-up. The use of artificial intelligence through ML modeling has great potential as a new approach to achieve higher levels of accuracy in diagnosing different types of cardiomyopathies (66–69).

## Author contributions

MW conceptualized, prepared, and wrote the manuscript and made the table. YX, SW, TZ, HC, YW, and RZ participated in providing documentation. CW reviewed, edited, and revised the manuscript. All authors have read and approved the final manuscript and assume full responsibility for its contents.
